# Comment on Pancreatitis in Type 1 Tyrosinemia

**DOI:** 10.4274/balkanmedj.2016.1209

**Published:** 2017-08-04

**Authors:** Hakim Rahmoune, Nada Boutrid, Mounira Amrane, Belkacem Bioud

**Affiliations:** 1 Department of Pediatrics, Setif University Hospital, Setif, Algeria; 2 Genetic and Nutritional Diseases Laboratory, Setif University, Setif, Algeria; 3 Department of Biochemistry, Setif University Hospital, Setif, Algeria

## To The Editor,

In their interesting, peculiar case report published in the May issue of the Balkan Medical Journal, Uçar et al. (1) reported a rare co-occurrence of acute pancreatitis with type 1 hereditary tyrosinaemia (HT1). This publication caught our attention regarding several relevant points.

First, in the patient details, the authors did not mention the results of any lumbar puncture, as the clinical course could reveal an acute viral encephalitis, especially post-mumps, that may be associated to pancreatitis.

Having normal urinary succinyl acetone is also very questionable after a long (6 months) period of nitisinone discontinuation. In fact, nitisinone 2-(2-nitro-4-trifluoro-methylbenzyol)-1,3 cyclohexanedione, NTBC) is a potent inhibitor of 4-hydroxyphenylpyruvate dioxygenase, an enzyme that is upstream of fumarylacetoacetase, and most patients present a rapid decrease in the concentrations of succinylacetone (2) when under NTBC.

On the other hand, acute pancreatitis is associated with a strong activation of the pro-inflammatory pathway (3). Local, as well as systemic inflammatory responses are independent of intra-acinar trypsinogen activation (4) and lead to the core inflammatory pathogenesis. A similar hyper-inflammatory state may be seen in HT1. Type 1 hereditary tyrosinaemia is caused by a deficiency of fumarylacetoacetate hydrolase, the enzyme responsible for the hydrolysis of fumarylacetoacetase. This latter metabolite, fumarylacetoacetase, displays mutagenic and apoptogenic activities and elicits an endoplasmic reticulum oxidative, inflammatory stress response (5).

Thus, treatment with NTBC would annihilate the fumarylacetoacetase accumulation, while complete NTBC withdrawal (as seen in this case) leads to a massive accumulation of fumarylacetoacetase.

We think that a possible aetiopathogenic, inflammatory cause of acute pancreatitis due to fumarylacetoacetase accumulating during NTBC withrawal might be considered in HT1 cases, and should enhance consideration of continuous enzyme inhibition with daily NTBC.
