# Supplementary material for: Comment on Pancreatitis in Type 1 Tyrosinemia
Source: Balkan Med J. 2017 Aug 4;34(4):380–1. doi: 10.4274/balkanmedj.2016.1209 (PMC5615974; doi:10.4274/balkanmedj.2016.1209)
Supplement: Related texts

# AUTHOR REPLY

**Authors:** Habibe Koç Uçar

**Affiliations:** 1 Department of Pediatrics, Çukurova University School of Medicine, Adana, Turkey

## To The Editor,

We thank Hakim Rahmoune and colleagues for their comments and questions. The clinical pictures of encephalitis and neurological crisis due to tyrosinemia type 1 are extremely different, and it is very hard to confuse these two neurological entities. The first one is an acute serious condition and mostly associated with the clinical findings of central nervous system like altered state of consciousness, convulsions and focal neurological deficits. In contrast, neurological crisis due to tyrosinemia type 1 is generally a sub-acute and gradually settled state associated with peripheral neuropathy, autonomous dysfunction.

Therefore, lumbar puncture was regarded as an unnecessary and invasive procedure for our patient's clinical statement. Neurological crisis was initially considered with clinical and laboratory findings, also discontinuation of 2-(2-nitro-4-trifluoro-methylbenzyol)-1,3 cyclohexanedione. As, the most prominent sign was abdominal pain and electromyography was normal; we had to evaluate the other possible causes which let us to the diagnosis of pancreatitis.

Urinary 5-delta-aminolevulinic acid and succinylacetone were studied in another center and the results were handed to us, these analyses can not be performed in our laboratory. As, succinylacetone is detectable in blood before urine, it is more accurate to determine it in blood. But, the clinical, laboratory and radiological findings pointed out acute pancreatitis without any suspicion.
